# Molecular analysis of cyst fluids improves the diagnostic accuracy of pre-operative assessment of pancreatic cystic lesions

**DOI:** 10.1038/s41598-021-81065-2

**Published:** 2021-02-03

**Authors:** Lena Haeberle, Martin Schramm, Wolfgang Goering, Lisa Frohn, Caroline Driescher, Werner Hartwig, Hubert-Karl Preissinger-Heinzel, Torsten Beyna, Horst Neuhaus, Katharina Fuchs, Verena Keitel-Anselmino, Wolfram Trudo Knoefel, Irene Esposito

**Affiliations:** 1 Institute of Pathology, Heinrich-Heine University and University Hospital of Duesseldorf, Moorenstr. 5, 40225 Duesseldorf, Germany; 2 Department of Surgery, Evangelisches Krankenhaus, Duesseldorf, Germany; 3 Department of Internal Medicine, Evangelisches Krankenhaus, Duesseldorf, Germany; 4 Clinic for Gastroenterology, Hepatology and Infectious Diseases, Heinrich-Heine University and University Hospital of Duesseldorf, Duesseldorf, Germany; 5 Department of Visceral, Thoracic and Pediatric Surgery, Heinrich-Heine University and University Hospital of Duesseldorf, Duesseldorf, Germany

**Keywords:** Cancer, Genetics, Molecular biology, Biomarkers, Gastroenterology, Medical research, Molecular medicine, Oncology

## Abstract

Pancreatic cystic lesions (PCL) are increasingly diagnosed. Endoscopic ultrasound fine-needle aspiration (EUS-FNA) cytology is often used for diagnostic confirmation but can be inconclusive. In this study, the role of molecular analyses in the pre-operative diagnostics of PCL is evaluated. Targeted Next Generation Sequencing (NGS) applied on cytology smears was retrospectively evaluated in a cohort of 37 resected PCL. Usefulness of NGS on fresh cyst fluids was tested in a prospective cohort of patients with newly diagnosed PCL (n = 71). In the retrospective cohort, cytology plus NGS displayed higher sensitivity (94.1% vs. 87.1%) and specificity (100% vs. 50%) than cytology alone for the detection of mucinous neoplasms. In the prospective cohort, sensitivity and specificity of conventional cytology alone were 54.2% and 100% for the detection of mucinous neoplasia and 50.0% and 100% for the detection of high-grade dysplasia, respectively. Adding NGS, all lesions which underwent histopathologic verification (12/71, 17%) could be classified without false positive or false negative results regarding the detection of mucinous neoplasm so far. NGS analysis of cfDNA in PCL fluids is feasible and can increase diagnostic accuracy in the detection of mucinous neoplasms compared to cytology alone. However, algorithms for the detection of high-risk lesions need further improvement.

## Introduction

Pancreatic cystic lesions (PCL) are increasingly diagnosed on imaging. The prevalence of incidental PCL on imaging is reported to be 2.6–13.5%^1–3^. Therefore, their work-up and management has become an important issue for clinicians and pathologists. PCL can range from benign to high-grade dysplastic lesions or even cancer, and the decision for or against surgery can be challenging^1–3^. Among resected PCL, intraductal papillary mucinous neoplasms (IPMN) are the most common pancreatic cystic neoplasms, followed by mucinous (MCN) and serous (SCN) cystic neoplasms^4–6^. Branch-duct IPMN pose a problem, as their risk for high-grade dysplasia is significantly lower compared to main-duct IPMN (4–48% in cysts with a diameter of at least 30 mm vs. 30–91% in main-duct IPMN with a main duct dilation of at least 5 mm^7–17^). While all patients with main-duct IPMN who are fit for surgery should undergo resection, absolute criteria for the resection of branch-duct IPMN include positive cytology for high-grade dysplasia or cancer, among other (radiological and clinical) criteria^7,18–20^. During standard diagnostics, endoscopic ultrasound fine needle aspiration (EUS-FNA) is recommended according to European and American guidelines for cases with unclear imaging results regarding the distinction between mucinous vs. non-mucinous neoplasm and benign vs. malignant lesion^7,21^, although other guidelines ascribe a more controversial role to EUS-FNA, only recommending it in very specific settings^22^. The reason for this controversy is the possibility of complications combined with the fact that the diagnostic accuracy of EUS-FNA conventional cytology is relatively low (55–59%)^23–25^. This is mostly due to low sensitivity, which is likely a result of low cellularity and gastrointestinal contamination of the aspirated material.

Traditionally, DNA image cytometry for the detection of aneuploidy and fluorescent in situ hybridization (FISH) can be applied as ancillary methods to cytologic specimens to increase the diagnostic accuracy for the detection of high-grade and malignant lesions^26–33^. In recent years, the exome of solid and cystic pancreatic neoplasms has been sequenced, leading to the identification of driver variants^34^. *KRAS* variants are frequently found both in IPMN and in MCN, whereas *GNAS* codon 201 variants are characteristic of IPMN and can be found in up to 2/3 of cases^35^. Pathogenic variants of *TP53*, and to a lesser extent, of *SMAD4*, *PIK3CA* and *CDKN2A* are associated with high-grade dysplasia^36,37^. Previous studies suggest that NGS-based analysis of PCL fluid can vastly support the identification of mucinous neoplasms and, partially, the detection of high-grade lesions^36,38^. However, the experience with these techniques in the context of PCL is still limited and results need to be further validated. The aim of this study was the evaluation of feasibility and diagnostic value of NGS-based molecular analyses in the pre-operative assessment of PCL.

## Results

### Retrospective cohort

**Figure 1:**
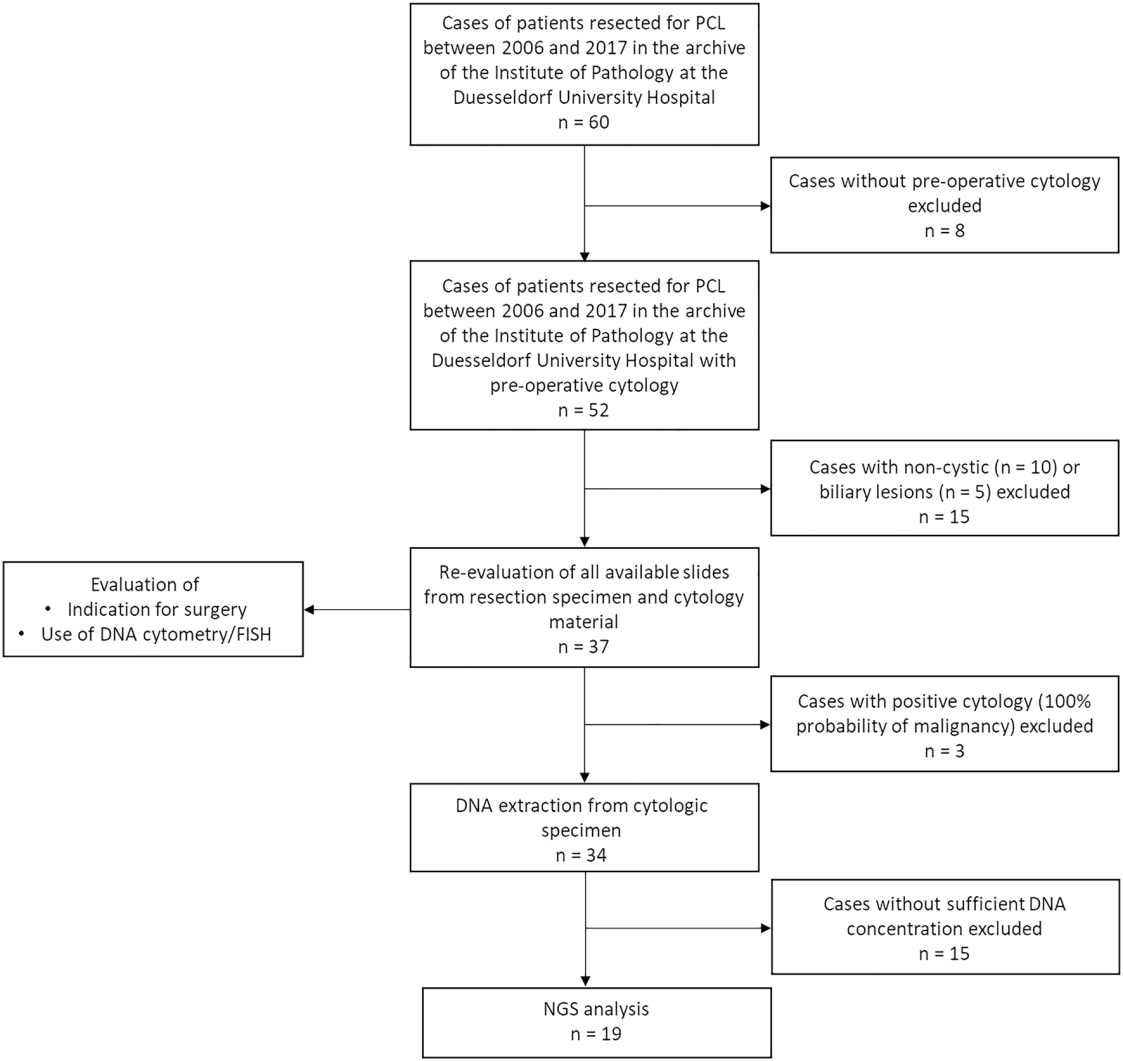
Workflow for the retrospective cohort. 37 cases of PCL with pre-operative cytology and post-operative histology were selected. All slides were re-evaluated according to current classifications. Targeted NGS of cytologic samples was performed and findings were interpreted in the context of pre-operative cytological and final post-operative diagnosis.

**Figure 2:**
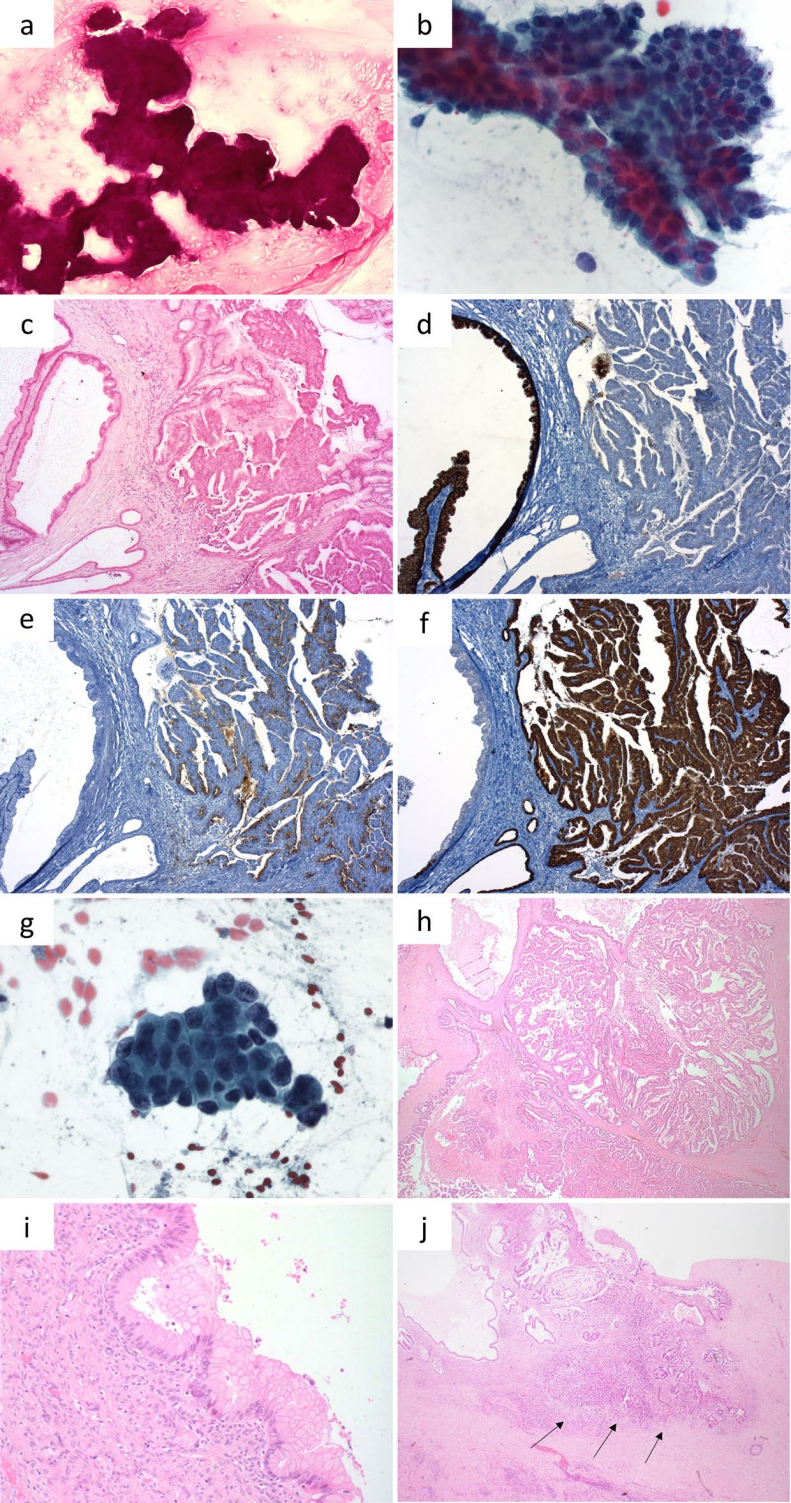
Exemplary cases from the retrospective (**A**–**F**) and the prospective cohort (**G**–**J**). (**A**–**F)** Retrospective case: branch-duct IPMN of mixed histologic type with low- and high-grade dysplasia. NGS revealed pathogenic mutations of *KRAS* and *TP53*. **A:** Mucinous background in cytology (PAS, 10 ×). (**B**) Papillary epithelia with atypia, suggesting mucinous neoplasm, possibly IPMN, in cytology (Papanicolaou, 40 ×). (**C**) Upon histology, areas with gastric differentiation and low-grade dysplasia (left) and areas with oncocytic differentiation and high-grade dysplasia (right) are seen (HE, 50 ×). (**D**) Gastric differentiation of low-grade area confirmed by MUC5C positivity (50 ×). (**E**,**F**) Oncocytic differentiation of high-grade areas confirmed by MUC1 and MUC6 positivity (50 ×). (**G**–**J**) Prospective case: MCN with high-grade dysplasia and small invasive adenocarcinoma. Pathogenic mutations of *KRAS* and *TP53* in NGS. (**G**) Cytology shows atypical mucinous epithelia with irregular nuclei (Papanicolaou, 40 ×). (**H**) Upon histology, a multicystic lesion is seen. Some areas are lined by one layer of mucinous epithelia with low-grade atypia (left), some show complex papillary projections with high-grade atypia (right) (HE, 20 ×). (**I**) Subepithelial ovarian-like stroma (HE, 400 ×). (**J**) Circumscribed invasive carcinoma (arrows) (HE, 20 ×).

Sixty patients resected for PCL between 2006 and 2017 were identified in the archive of the Institute of Pathology of the University Hospital Duesseldorf. Of these cases, 52 had received pre-operative EUS-FNA cytology. 10 of these cases (19.2%) were excluded because they turned out to be non-cystic and 5 cases (9.6%) because they turned out to be biliary lesions (n = 5; 9.6%) upon resection. The remaining 37 cases represent our retrospective cohort (Fig. 1). It consists of 28 IPMN (75.7%), two PDAC with cystic features (5.4%), one MCN with low-grade dysplasia (2.7%), one SCN (2.7%), one intraductal tubulo-papillary neoplasm (ITPN) (2.7%), one solid-pseudopapillary neoplasm (SPN) (2.7%), one pyloric gland adenoma (2.7%), one simple mucinous cyst and one inflammatory pseudocyst (2.7%) (Table 1 and Fig. 2A–F). In 7/37 cases (18.9%), DNA cytometry and/or FISH had been used as ancillary methods during the diagnostic process, and in 3 of them (8% of the entire collective), DNA cytometry had contributed to reach a correct diagnosis.

**Table 1:**
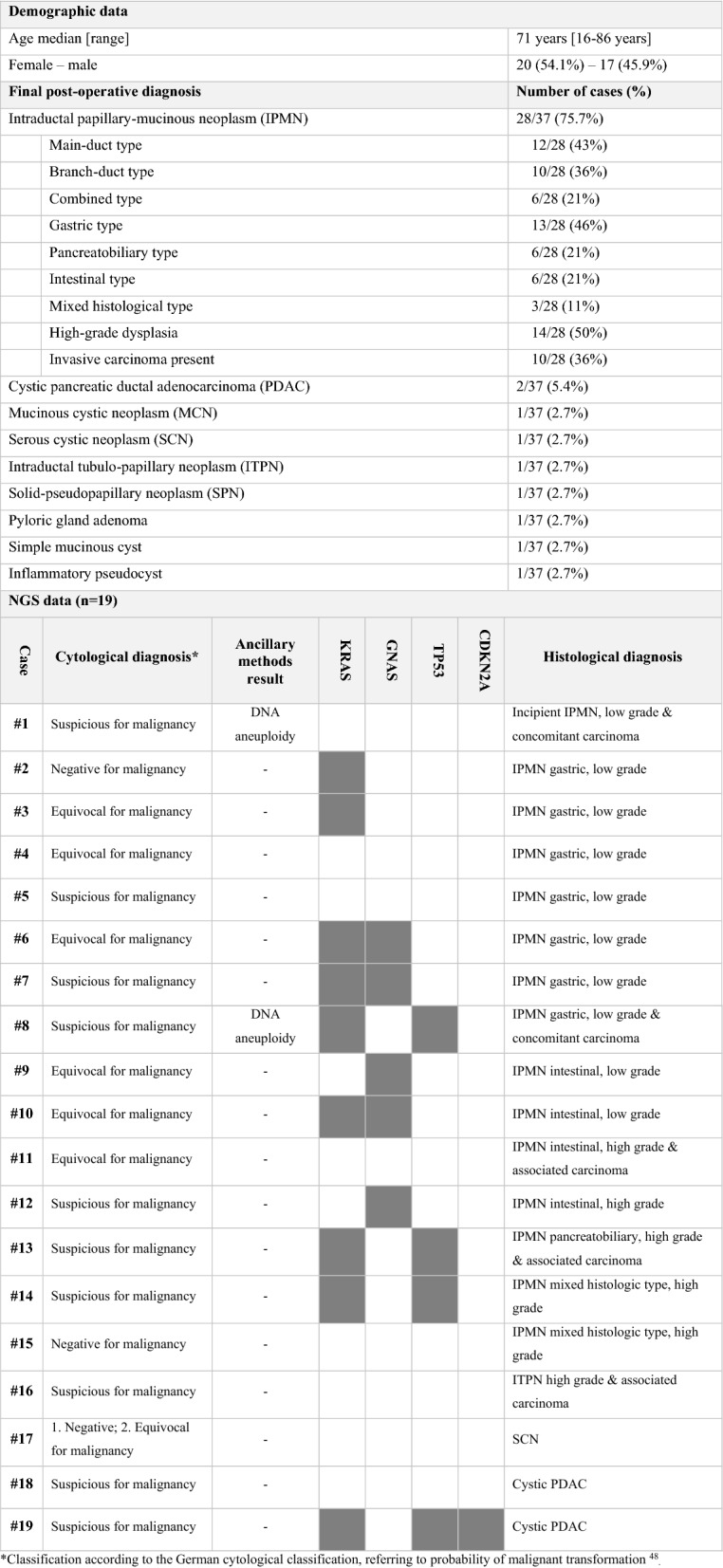
Retrospective cohort (n = 37).

*Classification according to the German cytological classification, referring to probability of malignant transformation^48^_._

Indication for surgery (i.e. diagnosis of a high-risk lesion: presence of high-grade dysplasia or frank carcinoma, main-duct IPMN, other potentially malignant lesions such as SPN) had been correctly set in 30/37 cases (81%), while in the remaining 7 (19%) surgery could have been avoided/delayed, as only benign or low-grade lesions were present upon evaluation of the resection specimen. All cases with ambiguous conventional cytology (*negative*, *equivocal* or *suspicious* category) were tested for an additional value of NGS retrospectively. This cohort comprised 19 cases, in which DNA of sufficient quality could be extracted (Fig. 1). In 11/19 cases (58%), at least one pathogenic variant was found, which would have been useful to obtain a clear-cut cytopathologic pre-operative diagnosis. In detail, 10/15 IPMN could have been diagnosed as mucinous neoplasms by the presence of *KRAS* and/or *GNAS* variant, whereas both non-mucinous lesions (one ITPN and one SCN) did not show variants either in *KRAS* or in *GNAS*. In addition, diagnosis of IPMN instead of a generic diagnosis of “mucinous neoplasms” could have been made in 5/15 IPMN cases (33.3%), in which a pathogenic GNAS variant was found. High-grade lesions could have been detected by presence of *TP53* and/or *CDKN2A* variant in NGS in 4/10 cases (40%). On the other hand, NGS never detected variants indicative for high-grade dysplasia in low-grade lesions. Altogether, in the retrospective cohort, NGS analysis would have been determinant to achieve diagnosis in 11/19 (57%), which had been reported as *suspicious* or *equivocal*, or even *negative* in one case (Table 1).

The sensitivity and specificity of conventional methods without NGS for the diagnosis of a mucinous neoplasm were 87.1% (95% CI (confidence interval) 70.2–96.4%) and 50% (95% CI 11.8–88.2%), respectively, with an overall accuracy of 81.1% (95% CI 64.8–92%). With NGS, the sensitivity, specificity, and accuracy for the detection of a mucinous neoplasm increased to 94.1% (95% CI 71.3–99.6%), 100% (95% CI 51.8–100%) and 88.9% (95% CI 51.8–99.7%), respectively. The sensitivity and specificity of conventional methods without NGS for the diagnosis of high-grade dysplasia were 85% (95% CI 62.1–96.8%) and 76.5% (95% CI 54.4–96%), respectively, with an overall accuracy of 83.3% (95% CI 67.2–93.6%). With NGS, there was a slight increase in sensitivity (88.9%; 95% CI 51.8–99.7%) for the detection of high-grade neoplasms, but no significant change in specificity or accuracy.

### Prospective cohort

**Figure 3:**
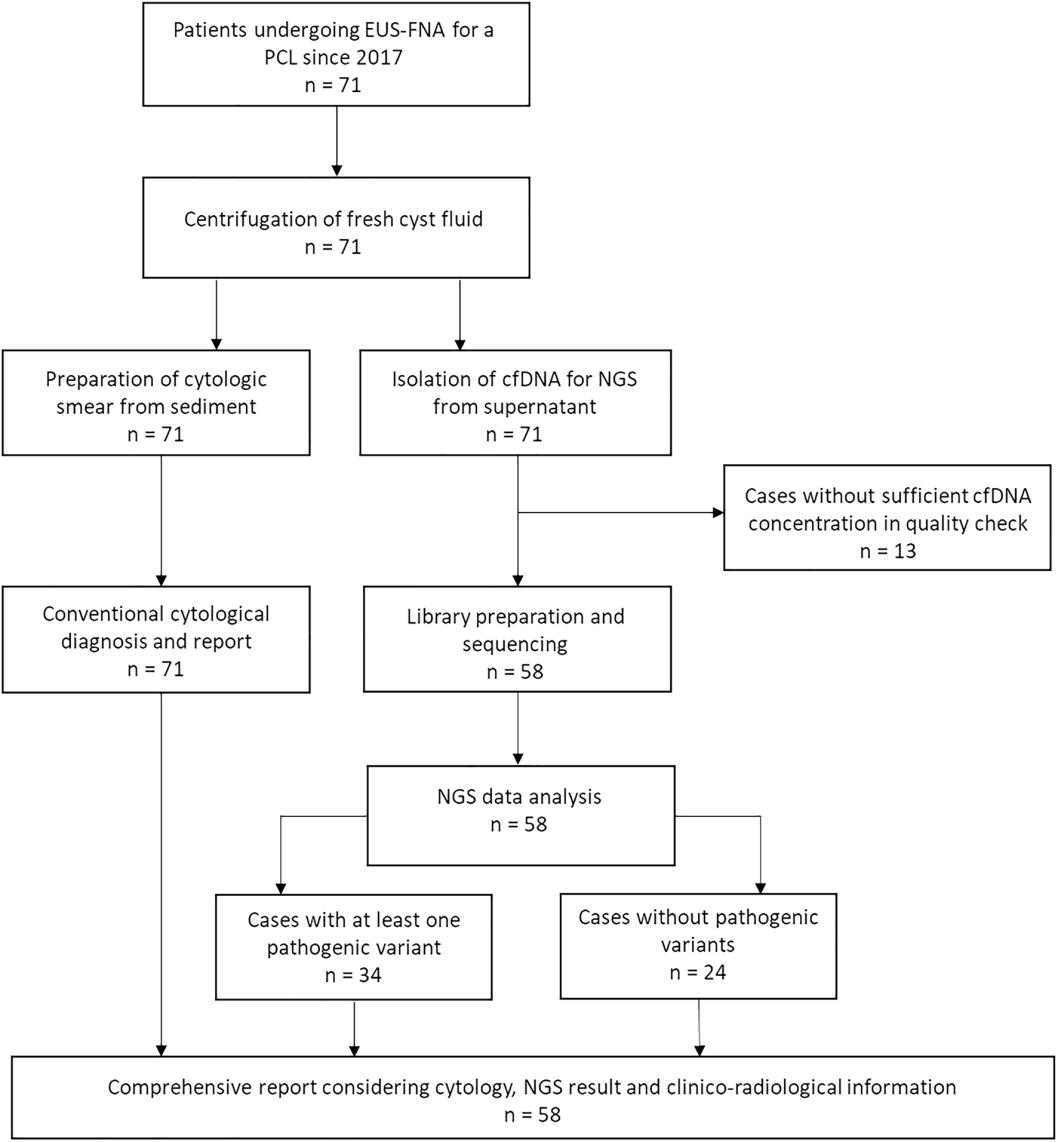
Workflow for the prospective cohort. FNA sample is centrifuged upon arrival, supernatant is collected. Cytological smears are prepared from sediment. Cytopathological diagnosis is performed and findings are reported. Simultaneously, cfDNA is isolated from supernatant. If sufficient cfDNA concentration is obtained, library is prepared and NGS is performed. Sequencing data are analyzed and a comprehensive report considering clinico-radiological information, conventional cytology results and sequencing data is prepared.

71 patients newly diagnosed with PCL who underwent preoperative EUS-FNA were included in the prospective cohort (Fig. 3). Of this prospective cohort, 12 patients (17%) later underwent biopsy or resection.

According to the 5-tiered German classification, 3/71 cases (4.2%) were ascribed to the category *not sufficient*, 56/71 cases (78.9%) were *negative for malignancy*, 5/71 cases (7%) were *equivocal* (“atypical, malignant cells cannot be excluded”), 3/71 cases (4.2%) were *suspicious for malignancy*, and 4/71 cases (5.6%) were *positive for malignancy*. The concentration and quality of the extracted DNA was sufficient for NGS in 58/71 cases (81.7%). At least one pathogenic variant was detected in 34/58 cases (58.6%) (Table 2 and S1). When comparing cfDNA samples with DNA extracted from smear samples, we found higher mean allelic frequencies (AF) of variants in the latter. For example, the mean AF of *KRAS was* 15.5% using cfDNA vs. 41.6% using DNA from smear; the mean AF for *GNAS* was 19.5% using cfDNA vs. 25.5% using DNA from smear.

**Table 2:**
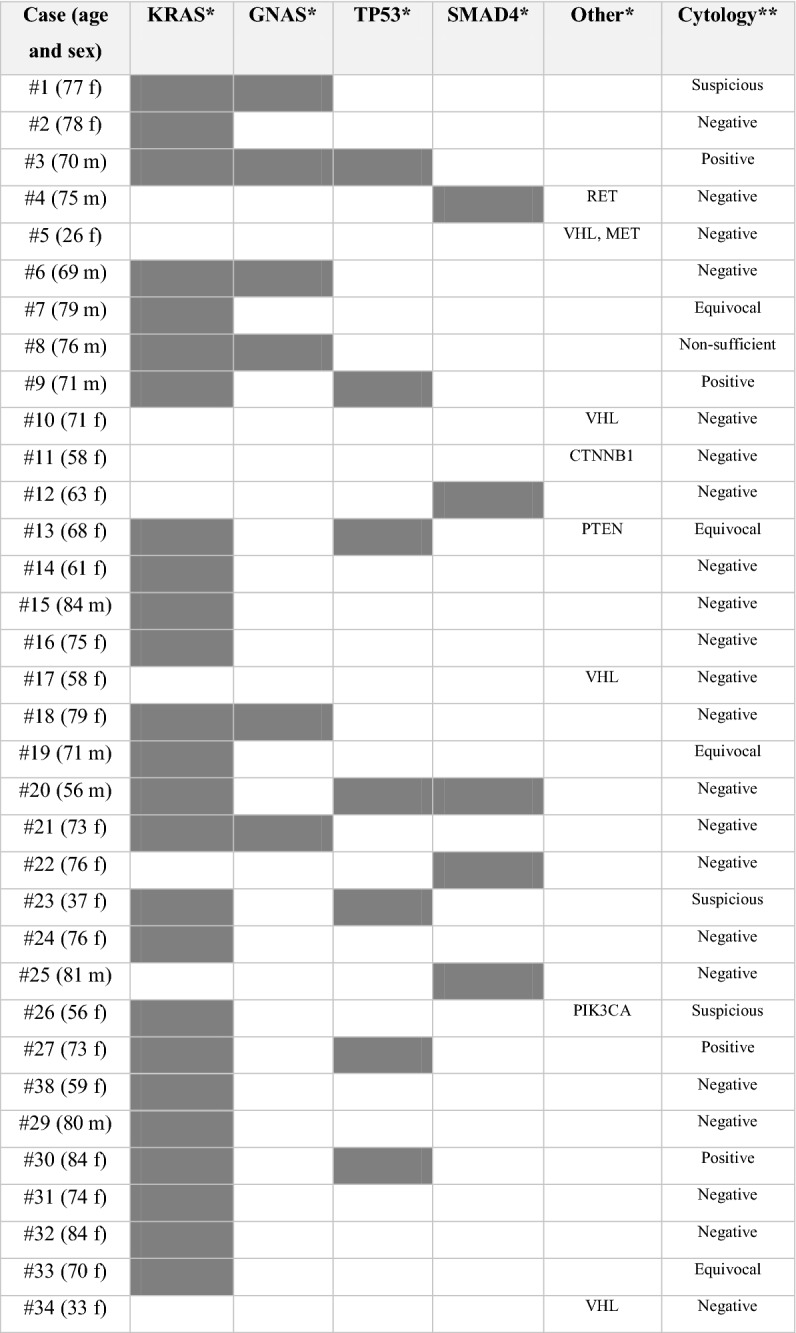
Overview of the prospective cohort cases with at least one pathogenic variant (n = 34).

*f* female, *m* male.

*In few cases, more than one pathogenic variant of the respective gene was found.

**Non-sufficient/negative/equivocal/suspicious/positive: Interpretation according to German classification^48^.

Thus far, 12/71 patients of the prospective cohort (17%) underwent diagnostic biopsy (n = 5, 7%) or surgical resection (n = 7, 10%). An overview of the cases with histopathological diagnosis is given in Table 3. In detail, one case was diagnosed as autoimmune pancreatitis type 2 upon biopsy, after *negative* cytology (no epithelial cells) and no pathogenic variants in NGS. Two further cases were diagnosed as PDAC upon biopsy; conventional cytology was *positive* and NGS revealed pathogenic variants of *KRAS* and *TP53*. In two other cases, PDAC was also diagnosed in biopsy; in both cases, conventional cytology was *negative* and NGS revealed a pathogenic variant of *KRAS*, at least indicative of mucinous neoplasm. Upon resection, a low-grade gastric-type IPMN was diagnosed in a case after *negative* cytology and detection of a pathogenic *KRAS* variant at NGS. The second resected case was classified as SCN upon final diagnosis, which fit the suspected diagnosis of clinicians and radiologists. In this case, conventional cytology was *not sufficient* and NGS revealed no pathogenic variant in the 50 genes covered by the Cancer Hotspot panel, which is generally consistent with SCN. The third resected case was diagnosed as PDAC with IPMN intestinal type, low grade, main-duct type, upon resection. In cytology, the case was *negative* and in NGS a pathogenic *SMAD4* variant was found, indicating high-grade neoplasm/malignancy. The fourth resected case was diagnosed as PDAC arisen from MCN, high grade. Upon conventional cytology, PAS-positive mucin and papillary cells with atypia had been seen, warranting the diagnosis *suspicious* in conventional cytology. Subsequently, NGS revealed pathogenic variants of *KRAS* and *TP53,* confirming high-grade mucinous neoplasm (Fig. 2G–J). The fifth resected case had *negative* conventional cytology, but showed a pathogenic *KRAS* variant, indicating mucinous neoplasm, and a pathogenic *SMAD4* and *TP53* variant, suggesting high-grade changes. Upon resection, the lesion was diagnosed as small multifocal PDAC derived from an IPMN gastric type, high grade, main-duct type. The sixth resected case was diagnosed as PDAC after *negative* conventional cytology and detection of a pathogenic variant of *KRAS*, which was at least indicative of mucinous neoplasm. The final case with surgical resection was diagnosed as mucinous cystic neoplasm low grade, after conventional cytology had been *equivocal* and NGS has revealed a pathogenic KRAS variant.

**Table 3:** Overview of the prospective cohort of cases with biopsy or surgical resection (n = 12).

Age/sex	Pathogenic variants	Cytology^a^	Histology
38 f	None	Negative	B: Autoimmune pancreatitis type 2
73 f	KRAS, TP53	Positive	B: Invasive adenocarcinoma
84 f	KRAS, TP53	Positive	B: Invasive adenocarcinoma
80 m	KRAS	Negative	B: Invasive adenocarcinoma
84 m	KRAS	Negative	B: Invasive adenocarcinoma
75 f	KRAS	Negative	S: IPMN gastric LG
58 f	None	Non-sufficient	S: SCN
81 m	SMAD4	Negative	S: Invasive adenocarcinoma and IPMN intestinal LG
37 f	KRAS, TP53	Suspicious	S: Invasive adenocarcinoma arisen from MCN HG
56 m	KRAS, TP53, SMAD4	Negative	S: Invasive adenocarcinoma arisen from IPMN gastric HG
74 f	KRAS	Negative	S: Invasive adenocarcinoma
71 m	KRAS	Equivocal	S: MCN LG

*B* biopsy, *S* surgical resection, *f* female, *m* male, *HG* high grade, *IPMN* intraductal papillary mucinous neoplasm, *LG* low grade, *MCN* mucinous cystic neoplasm, *SCN* serous cystic neoplasm.

^a^Non-sufficient/negative/equivocal/suspicious/positive: interpretation according to German classification^48^.

**Figure 4:**
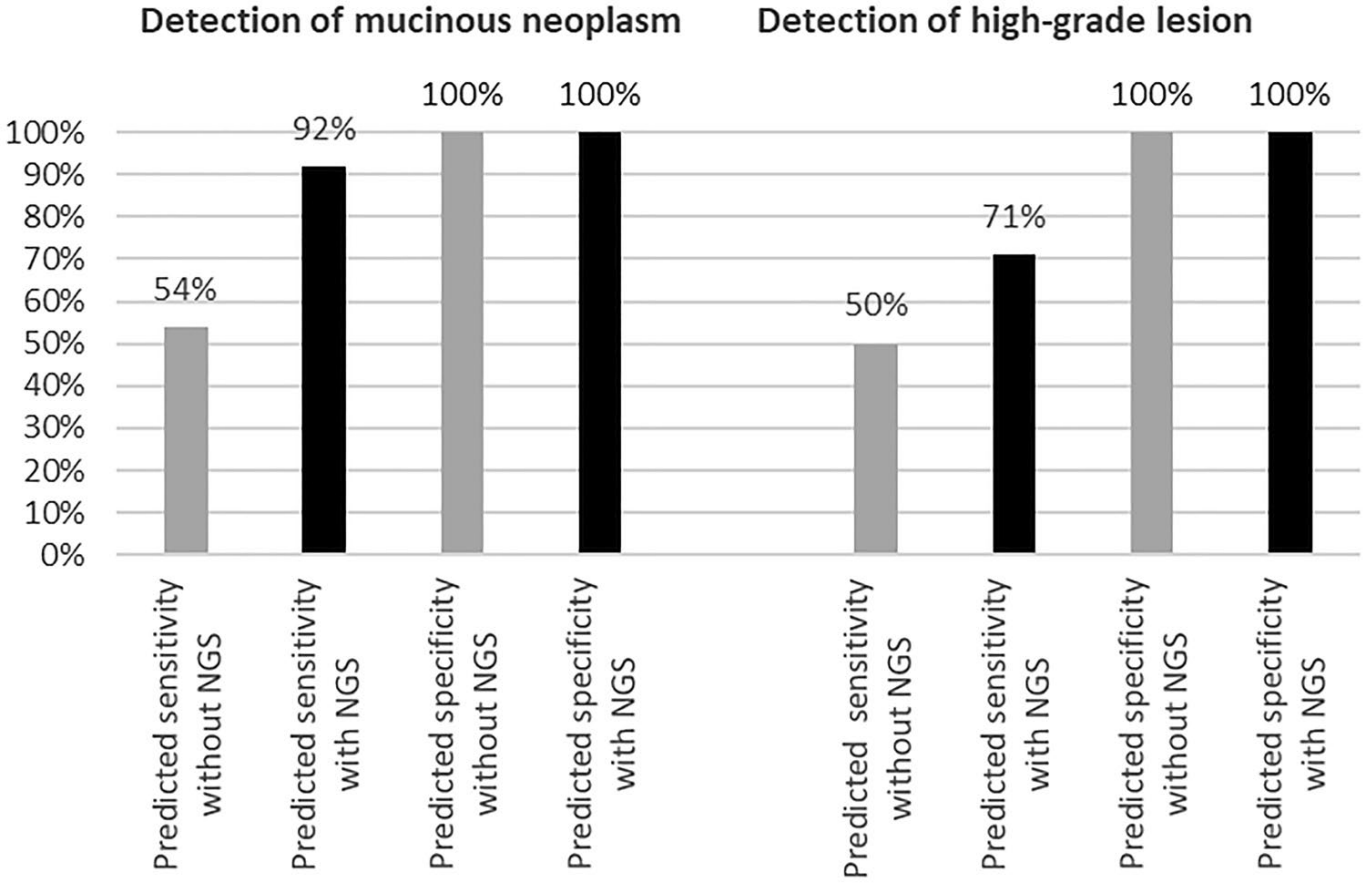
Predicted improvement of sensitivity and specificity in the detection of mucinous neoplasms and high-grade neoplasms in cytological samples via NGS in the prospective cohort.

Since only few patients in the prospective cohort received a final histopathological diagnosis (resection or biopsy), not only cases with final histopathological diagnosis, but also cases with unequivocal cytological result and/or unequivocal clinico-radiological findings were considered to calculate a prediction for the sensitivity and specificity of NGS in the prospective cohort. For example, a case with *positive* result in conventional cytology (high-grade mucinous neoplasm unequivocally diagnosed via conventional cytology) and pathogenic variants of *KRAS* and *TP53* in NGS was determined true positive for the detection of mucinous neoplasia and for the detection of high-grade neoplasia. A case with clinically evident chronic pancreatitis, unequivocal diagnosis of pseudocyst in radiology, and detection of no pathogenic variant in NGS was determined true negative for the detection of mucinous neoplasia and the detection of high-grade dysplasia. Using these cases (n = 36), predicted sensitivity and specificity of conventional cytology alone were 54.2% (95% CI 32.8–74.5%) and 100% (95% CI: n.a.) for the detection of mucinous neoplasm (accuracy: 94.4%; 95% CI 81.3–99.3%), and 50.0% (95% CI 41.9–91.6%) and 100% (95% CI: n.a.), respectively, for the detection of high-grade dysplasia (accuracy: 80.0%; 95% CI 63.1–91.6%). For conventional cytology plus NGS analysis, the predicted sensitivity and specificity were 91.7% (95% CI 73.0–99.0%) and 100% (95% CI: n.a.) for the detection of mucinous neoplasm (accuracy: 94.4%; 95% CI 81.3–99.3%), and 71.4% (95% CI 41.9–91.6%) and 100% (95% CI: n.a.) for the detection of high-grade dysplasia (accuracy: 88.9%; 95% CI 73.9–96.9%) (Fig. 4). Of course, these results are considerably limited by the low number of cases with histologically confirmed diagnosis and therefore need to be verified after a longer follow-up interval.

## Discussion

In this work, we show that targeted NGS can increase sensitivity of EUS-FNA cytology in the pre-operative detection of mucinous neoplasms compared to cytology alone. This can be explained by the fact that pathogenic variants indicating mucinous neoplasia (*KRAS* and *GNAS*) are detectable via NGS even in cases with suboptimal diagnostic material (artifacts, low cellularity or no cellular component at all). The prevalence of pathogenic *KRAS* or *GNAS* variants is high in mucinous neoplasm (approximately 2/3 of mucinous neoplasms contain a pathogenic variant of either *KRAS*, *GNAS* or both ^38^), making *KRAS* and *GNAS* variants reliable molecular markers.

The detection of high-grade dysplasia remains problematic, indicating the general lack of reliable gold standard molecular markers of high-grade dysplasia so far. The relatively low sensitivity in the detection of high-grade lesions, even when combining conventional cytology and NGS, is largely the result of the fact that pathogenic variants of *TP53/CDKN2A/SMAD4* are often not detected in pancreatic cyst fluids, even in those of histopathologically confirmed high-grade lesions. To a much lesser extent, mucinous lesions without pathogenic KRAS/GNAS mutations also exist. However, the above-mentioned markers were used as molecular surrogates for high-grade neoplasia and mucinous neoplasia, respectively, as more sensitive markers are currently not yet established.

In our retrospective cohort, of 10 cases with histologically confirmed high-grade dysplasia only 4 (40%) harbored pathogenic variants of *TP53* or other genes usually associated with high-grade dysplasia (Table 1). On the other hand, pathogenic variants of genes associated with high-grade dysplasia were never found in benign or low-grade lesions, accounting for a specificity of 100%. This is in line with results published by Rosenbaum and colleagues, who reported a specificity of 100% and a sensitivity of 45.8% for the detection of high-grade dysplasia in their series, while Singhi and colleagues found that the combination of *KRAS/GNAS* variants and *TP53/PIK3CA/PTEN* variants had a 100% specificity and 89% sensitivity for the detection of advanced neoplasia^36,37^. Accordingly, negative results in NGS alone cannot be used to spare patients from surgery, as the absence of variants does not allow the exclusion of a high-grade lesion. However, the presence of variants associated with high-grade dysplasia should support the decision for surgery, especially if clinico-radiological features are inconclusive.

Past studies^36–39^ and data from our retrospective and prospective cohorts show that NGS analyses can be performed successfully on cytology smears or even cell-free cyst fluid. When comparing cfDNA samples with DNA extracted from smear samples, we found higher mean allelic frequencies of gene variants in the smear samples, probably due to higher cellularity. However, cyst fluid analysis allowed to extract sufficient DNA in a significantly higher number of cases than extraction from smear (81.7% vs 55.9%, p = 0.005). This difference can be at least partially explained by technical considerations. First, extraction methods were different in the two collectives. The GeneRead DNA FFPE Kit is routinely used in our lab for isolation of DNA from formalin-fixed paraffin embedded (FFPE) samples and, skipping the deparaffinization step, for other samples containing intact fixed cells. Its main advantage is the reversion of crosslinks within the DNA and the removal of deaminated cytosine residues, both induced by formalin-fixation. Isolation of cfDNA diluted within body fluids is facilitated by specialized kits. The lower success rate of DNA extractions from smears compared to cfDNA from FNAs could be additionally explained by the longer storage time of many smear samples used in our validation cohort, whereas cfDNA from FNAs were routinely extracted immediately upon arrival. Therefore, combining conventional cytology with simultaneous cfDNA fresh cyst fluid analysis increases the chances to achieve a more accurate diagnosis, even in case of low cellularity.

FNB (fine-needle biopsy) of PCL, especially using a through-the-needle microforceps, represents an increasingly used technique which allows for histological sampling of tissue from the cyst wall, and may outperform FNA regarding diagnostic material yield and accuracy^40^. However, microforceps biopsy seems to be mainly superior in the diagnosis of specific cystic lesions, while its performance regarding the detection of mucinous and high-grade lesions seems comparable to that of FNA-based cyst fluid analyses including NGS^41^. Additionally, cfDNA from cyst fluid reflects changes regarding the whole lesion, while biopsies are always spatially limited and can only reflect part of a lesion.

Although only cases with clear clinico-radiological features (e.g. worrisome/high-grade features in cross-sectional imaging) were used to determine the diagnostic accuracy of NGS, the main limitation of our study resides in the low number of patients (12 of 71, 17%) who received final histopathological diagnosis in our prospective cohort. In fact, the accuracy of cross-sectional imaging methods in diagnosing the type of PCL is controversial, and both the distinction between mucinous and non-mucinous lesions and of low- and high-grade lesions are prone to bias^42^. On the other side, this situation reflects the application of current guidelines on the treatment of PCL and the clinical reality, in which patients do not receive biopsy and/or surgery either because it is not considered necessary after multidisciplinary discussion or because of contra-indications like co-morbidities. Performing NGS analysis may therefore be of help in supporting the clinical decision, as indicated by our results.

In conclusion, past studies and our data show that NGS analysis of PCL fluid is feasible using cell-free cyst fluid and is a useful tool that can increase sensitivity of cytological analysis regarding the detection of mucinous neoplasm, performing better than traditional ancillary methods and independently from the cellular content of the aspirates. As a critical point in the management of patients with PCL is the identification of high-grade changes, current studies have focused on new marker candidates to distinguish high-grade from low-grade dysplasia in pancreatic cyst fluids. For this purpose, methylation analysis of selected gene panels^43^ as well as the analysis of the cyst fluid miRNAome^44,45^ have been proposed in addition to the above-mentioned markers for malignant transformation. In the future, longitudinal retrospective studies—preferably including a large fraction of patients who underwent surgery and therefore have a confirmed final diagnosis—are needed to evaluate the role of novel markers for high-grade features as well as to further consolidate the role of NGS analyses in the pre-operative management of PCL.

## Methods

### Selection of patient cohorts

All cases of patients who were resected for PCL in the time period 2006–2017 were retrieved from the archive of the Institute of Pathology of the University Hospital of Duesseldorf, Germany, and re-evaluated according to current classification and nomenclature^46,47^. Cases with pre-operative EUS-FNA cytology were selected for further analysis. Cases which turned out non-cystic or of biliary origin upon resection were excluded. The remaining cases were used to assess the value of NGS analysis of cytology smears in the preoperative assessment of PCL, compared with conventional cytopathology and, where applicable, with conventional ancillary studies (DNA image cytometry, Fluorescent in situ Hybridization, FISH) (Fig. 1).

Patients newly diagnosed with PCL who underwent preoperative EUS-FNA represent our prospective cohort. In the prospective cohort, NGS-based analysis of cell-free DNA (cfDNA) extracted from the cyst fluid was integrated in routine diagnostic procedures (Fig. 3). Decision for surgery or follow-up was made according to current guidelines and after discussion in interdisciplinary tumor conferences.

### Cytopathology

#### Retrospective cohort

Immediately after EUS-FNA, smears were prepared from the aspirate and fixed with alcohol-spray (Merckofix; Merck KGaA, Darmstadt, Germany). The smears were stained according to Papanicolaou. If enough material was available, Periodic Acid Schiff (PAS) stain for detection of mucins was applied to one of the smears. The cytomorphologic findings were classified according to the probability of malignant transformation according to the German classification as follows: *Negative* (i.e. inflammation, benign cyst component), *equivocal* (atypical cells present, probability of malignant transformation approximately 30%), *suspicious* (probability of malignant transformation approximately 70%), *positive* (malignant transformation) or *not sufficient* (i.e. cell-free cyst content or only gastric/enteric contaminants present)^48^. Equivocal or suspicious smears were further analyzed with either DNA image cytometry or FISH (see below) whenever possible to exclude or confirm malignant transformation. If more than one slide was available, slides with the highest number of neoplastic cells were selected for NGS analysis.

#### Prospective cohort

Native pancreatic cyst fluids were centrifuged at 500×*g* (1800 rpm) for 5 min. Depending on the amount of the pellet, 3 smears or cytospins were prepared, alcohol-spray-fixed and stained according to Papanicolaou and with PAS stain for microscopic evaluation. Cytomorphologic findings were classified according to the above-mentioned 5-tiered German classification^48^. The supernatant with cfDNA was stored at − 20 °C for further molecular analysis.

### DNA image cytometry

Re-staining according to Feulgen and measurement of the nuclear DNA contents with a computer-based image analysis system (Motic, Xiamen, China) were applied to one of the smears as previously described^26,49^. After internal calibration with 30 reference nuclei (i.e. normal epithelial cells, granulocytes), the DNA content of approx. 300 manually selected nuclei was measured per specimen for detection of DNA aneuploidy. Proof of DNA aneuploidy (stem-line or single cell aneuploidy) indicates a malignant transformation of the analyzed cells. DNA stem-line aneuploidy was detected if the modal value of a stem-line was outside normal or euploid-polyploid values: < 1.80c or > 2.20c and < 3.60c or > 4.40c (c = DNA content). Single-cell aneuploidy corresponds to the detection of at least one cell in the specimen that had a DNA content > 9c. All technical instruments, all software used and guidelines for diagnostic interpretation and quality assurance met the standard requirements of the consensus reports of the European Society for Analytical Cellular Pathology^50–53^.

### Fluorescence in-situ hybridization (FISH)

Upon indication, the UroVysion multicolour FISH probe (Abbott/Vysis, Downers Grove, IL, USA) was used on pre-stained routine smears. The UroVysion multicolour-FISH probe combines centromeric probes for chromosomes 3, 7 and 17 and a locus-specific probe for the region 9p21. The processing of the smears, hybridization procedure and microscopic evaluation (Zeiss Axio Imager M1 fluorescence microscope; Zeiss, Goettingen, Germany) were performed as previously described^49,54^. At least 25 and a maximum of 60 atypical cells (identified by assessing nuclear enlargement, irregular shape, patchy staining with 4′,6-diamidine-2-phenylindole dihydrochloride (DAPI)) were evaluated. Aneusomy, corresponding to malignant transformation, was defined as the presence of > 6 cells with gain in two or more of the four samples. Tetrasomy, defined as the presence of 4 signals of at least 3 samples, was not considered abnormal^49^.

### Extraction and quantification of DNA from smears (retrospective cohort) and pancreatic cyst fluid (prospective cohort) for amplicon-based massive parallel sequencing

DNA from cells from smear preparations was extracted using GeneRead DNA FFPE Kit (Qiagen, Hilden, Germany) following manufacturer’s advices. CfDNA from pancreatic cyst fluid was extracted using QIAamp MinElute ccfDNA Kit (Qiagen, Hilden, Germany) following manufacturer’s recommendations. Extracted DNA was first quantified by Qubit dsDNA BR Assay Kit (Thermofisher, Darmstadt, Germany). Subsequently, quantitative PCR (qPCR) was performed employing a custom primer assay (HML-2 for: 5′-AAACGCCAATCCTGAGTGTC-3′; HML-2 rev: 5′-CATAGCTCCTCCGATTCCAT-3′) directed against a subset of long terminal repeats (LTRs) from HML 2 human endogenous retroviruses with Power SYBR Green PCR Master Mix on a StepOnePlus Real-Time PCR System (both Thermofisher, Darmstadt, Germany).

### Library preparation and massive parallel sequencing

Library preparation was carried out using Ion AmpliSeq Library Kit 2.0 and Ion AmpliSeq Cancer Hotspot Panel v2 with 10 ng of amplifiable DNA following manufacturer’s recommendations. Ion Xpress Barcode Adapters Kits were utilized for barcoding libraries. Afterwards, libraries were quantified by qPCR using Ion Library TaqMan Quantitation Kit on a StepOnePlus Real-Time PCR System and were compiled equimolarly for subsequent sequencing reaction. Massive parallel sequencing was conducted on an Ion S5 System using the Ion 520 and Ion 530 Kit-OT2 with an Ion 530 Chip. Primary data analyses were performed by Ion Torrent Suite Software. For variant annotation, generated Binary Alignment Map (BAM) files were uploaded to and analysed by Ion Reporter Software using recommended analysis parameter for the Ion AmpliSeq Cancer Hotspot Panel v2. Detected variants were examined using the Integrative Genomics Viewer (IGV)^55,56^. All reagents and software were from Thermofisher (Darmstadt, Germany).

### Data interpretation

The indication for surgery in the retrospective cohort was considered correct if—upon resection—a diagnosis of high-grade dysplasia, invasive carcinoma or potentially malignant neoplasm (e.g. SPN) was made.

‘Positive diagnosis’ in conventional cytopathology was defined as a 100% probability of malignancy, according to the definition of the 5-tiered German classification for cytopathological diagnosis^48^.

‘Mucinous lesion’ based on NGS was defined as PCL with pathogenic *KRAS* and/or *GNAS* mutation. ‘IPMN’ based on NGS was defined as PCL with pathogenic GNAS mutation. ‘High-grade dysplasia/lesion’ based on NGS was defined as lesion with pathogenic *TP53* and/or *CDKN2A* and/or *SMAD4* mutation.

### Statistical analysis

Calculation of sensitivity, specificity and accuracy was performed using MedCalc calculators online (https://www.medcalc.org/calc/diagnostic_test.php; MedCalc Software, Ostend, Belgium).

### Ethical approval

The study was performed in accordance with the Declaration of Helsinki statement for medical research involving human subjects. The use of human samples was approved by the local ethics committee at the Heinrich Heine University and University Hospital of Duesseldorf, Germany (study no. 3821, amendment of June 18th, 2019). Informed consent to participate and informed consent for publication were obtained according to the guidelines and instructions by the aforementioned ethics committee. All data is stored in a pseudonymized manner according to the guidelines and instructions by the aforementioned ethics committee.

## Supplementary Information

Supplementary Information
