# Supplementary material for: Molecular analysis of cyst fluids improves the diagnostic accuracy of pre-operative assessment of pancreatic cystic lesions
Source: Sci Rep. 2021 Feb 3;11:2901. doi: 10.1038/s41598-021-81065-2 (PMC7858638; doi:10.1038/s41598-021-81065-2)
Supplement: Supplementary file 1 — Supplementary Information [file 41598_2021_81065_MOESM1_ESM.docx]

**Supplementary Table S1. Pathogenic variants identified in the prospective cohort (n=71; NGS analysis of n=58).**

| **Variant** | **No. (%)** |
| --- | --- |
| **KRAS (NM_033360.3)** | **25/58 (43.1%)** |
| c.35G>T (p.Gly12Val) | 10/58 (17.2%) |
| c.35G>A (p.Gly12Asp) | 9/58 (15.5%) |
| c.34G>C (p.Gly12Arg) | 5/58 (8.6%) |
| c.183A>C (p.Gln61His) | 1/58 (1.7%) |
|  |  |
| **GNAS (NM_000516.5)** | **7/58 (12.1%)** |
| c.601C>T (p.Arg201Cys) | 5/58 (8.6%) |
| c.602G>A (p.Arg201His) | 2/58 (3.4%) |
|  |  |
| **TP53 (NM_000546.5)** | **7/58 (12.1%)** |
| c.524G>A* (p.Arg175His) | 2/58 (3.4%) |
| c.811G>T (p.Glu271Ter) | 1/58 (1.7%) |
| c.818G>A (p.Arg273His) | 1/58 (1.7%) |
| c.832C>A (p.Pro278Thr) | 1/58 (1.7%) |
| c.844C>T (p.Arg282Trp) | 1/58 (1.7%) |
| c.1024C>T (p.Arg342Ter) | 1/58 (1.7%) |
|  |  |
| **SMAD4 (NM_005359.5)** | **5/58 (8.6%)** |
| c.767A>T (p.Gln256Leu)** | 3/58 (5.2%) |
| c.1060G>C (p.Val354Leu)** | 1/58 (1.7%) |
| c.1081C>T (p.Arg361Cys) | 1/58 (1.7%) |
|  |  |
| **VHL (NM_000551.3)** | **4/58 (6.9%)** |
| c.257C>T (p.Pro86Leu) | 1/58 (1.7%) |
| c.291delC (p.Tyr98fs) | 1/58 (1.7%) |
| c.343C>T (p.His115Tyr) & c.407T>C (p.Phe136Ser) (biallelic) | 1/58 (1.7%) |
| c.464-1G>T (splicesite_5) | 1/58 (1.7%) |
|  |  |
| **CTNNB1 (NM_001904.3)** | **1/58 (1.7%)** |
| c.94G>T (p.Asp32Tyr) | 1/58 (1.7%) |
|  |  |
| **PIK3CA (NM_006218.3)** | **1/58 (1.7%)** |
| c.1625A>T (p.Glu542Val) | 1/58 (1.7%) |
|  |  |
| **PTEN (NM_000314.6)** | **1/58 (1.7%)** |
| c.203A>G (p.Tyr68Cys) | 1/58 (1.7%) |
|  |  |
| **RET (NM_020975.4)**** | **1/58 (1.7%)** |
| c.1946C>T (p.Ser649Leu) | 1/58 (1.7%) |
|  |  |
| **MET (NM_001127500.2)**** | **1/58 (1.7%)** |
| c.2962C>T (p.Arg988Cys) | 1/58 (1.7%) |

*One sample includes additional TP53 c.818G>A variant with low allelic frequency.
**Predicted pathogenic *in silico* (COSMIC/FATHMM).
